# HHV-6A Infection of Papillary Thyroid Cancer Cells Induces Several Effects Related to Cancer Progression

**DOI:** 10.3390/v15102122

**Published:** 2023-10-19

**Authors:** Stefania Mardente, Maria Anele Romeo, Angela Asquino, Agnese Po, Maria Saveria Gilardini Montani, Mara Cirone

**Affiliations:** 1 Department of Experimental Medicine, Sapienza University, 00161 Rome, Italy; stefania.mardente@uniroma1.it (S.M.); mariaanele.romeo@uniroma1.it (M.A.R.); angela.asquino@uniroma1.it (A.A.); mariasaveria.gilardinimontani@uniroma1.it (M.S.G.M.); 2 Department of Molecular Medicine, Sapienza University, 00161 Rome, Italy; agnese.po@uniroma1.it

**Keywords:** HHV6A, thyroid cancer, papillary cancer, EMT, microRNAs, IL6

## Abstract

Recent studies have shown that thyrocytes are permissive to HHV-6A infection and that the virus may contribute to the pathogenesis of autoimmune thyroiditis. Thyroid autoimmune diseases increase the risk of papillary cancer, which is not surprising considering that chronic inflammation activates pathways that are also pro-oncogenic. Moreover, in this condition, cell proliferation is stimulated as an attempt to repair tissue damage caused by the inflammatory process. Interestingly, it has been reported that the well-differentiated papillary thyroid carcinoma (PTC), the less aggressive form of thyroid tumor, may progress to the more aggressive follicular thyroid carcinoma (FTC) and eventually to the anaplastic thyroid carcinoma (ATC), and that to such progression contributes the presence of an inflammatory/immune suppressive tumor microenvironment. In this study, we investigated whether papillary tumor cells (BCPAP) could be infected by human herpes virus-6A (HHV-6A), and if viral infection could induce effects related to cancer progression. We found that the virus dysregulated the expression of several microRNAs, such as miR-155, miR-9, and the miR-221/222 cluster, which are involved in different steps of carcinogenesis, and increased the secretion of pro-inflammatory cytokines, particularly IL-6, which may also sustain thyroid tumor cell growth and promote cancer progression. Genomic instability and the expression of PTEN, reported to act as an oncogene in mutp53-carrying cells such as BCPAP, also increased following HHV-6A-infection. These findings suggest that a ubiquitous herpesvirus such as HHV-6A, which displays a marked tropism for thyrocytes, could be involved in the progression of PTC towards more aggressive forms of thyroid tumor.

## 1. Introduction

Thyroid cancer represents one of the most frequent endocrine malignancies, with an incidence that has been increasing during the past decades. The majority of thyroid tumors that originate from thyroid epithelial follicular cells may be classified into different types: well-differentiated papillary thyroid carcinoma (PTC), follicular thyroid carcinoma (FTC), and anaplastic thyroid carcinoma (ATC) [1]. The latter is less differentiated, more aggressive, and refractory to therapies thyroid tumor characterized by recurrence and low survival rates. It has been reported that PTC may progress to ATC, a process called anaplastic transformation, and, in some cases, PTC and ATC may co-exist within the same tumor [2]. Although the mechanisms leading to thyroid tumor progression have not been completely elucidated, the nature of tumor microenvironment seems to play an important role. Indeed, thyroid tumors are infiltrated by lymphocytes and by other inflammatory cells that, as for other cancers, may strongly influence prognosis and response to treatments [3]. Several studies have reported a positive correlation between thyroid tumor aggressiveness and the presence of tumor-associated macrophages (TAMs) [4,5] or mast-cells [6] within the tumor microenvironment. Pro-tumorigenic effects may be induced by regulatory T cells, attracted to thyroid tumors by the release of chemokines. The activity of regulatory T cells is also sustained by cytokines produced by tumor cells as well as by other dysfunctional immune cells infiltrating tumors, facilitating tumor immune evasion and progression [7]. Interestingly, the onset of papillary cancer has been reported to occur more often in the context of the inflammatory background characterizing autoimmune thyroiditis such as Hashimoto thyroiditis (HT). Whether autoimmune thyroiditis represents a real risk factor for papillary cancer or if the two pathologies arise independently remains an open question. However, morphological, immunohistochemical and molecular profiles seem to favor the first hypothesis over the second [8]. Indeed, as for other organs, chronic inflammation characteristic of thyroiditis, could favor the development of thyroid cancer. This may be due to the fact that in response to inflammation-induced damage, expression of tumor suppressor genes or oncogenes [9] may change, and their dysregulated expression may be also detected as danger signals by the immune cells, perpetuating or even exacerbating the inflammatory process [10]. Previous studies have shown that HT shares with papillary cancer some molecular profiles such as, for example, the re-arrangement of RET proto-oncogene (RET/PTC oncogene) [11], BRAF mutation [12], and phosphatidylinositol 3-kinase (PI3k) pathway activation [13,14].

Moreover, micro-RNAs (miRNAs), such as miR-221 and miR-222, have been found to be dysregulated in inflammatory pathologies as well as in cancer [15,16]. Regarding thyroid, miRNAs may be considered biomarkers for the diagnosis, treatment, and prognosis of tumors. Particularly important is the miR-221/miR-222 cluster, involved in the thyroid hormone signaling pathway and other pathways correlated to the onset and progression of thyroid cancer [17]. The upregulation of miR-221/miR-222, in association with that of miR-146b-5p, has also been shown to be involved in thyroid cancer recurrence. In addition, miR-9, a transcriptional target of c-Myc, has been found to be over-expressed in ATC tissues and correlated with aggressiveness and epithelial mesenchymal transition (EMT), namely, the acquisition of mesenchymal features by epithelial cells [18].

Ubiquitous viruses such as herpes simplex 1 (HSV) are frequently detected in thyroid cancer tissues, and it has been reported that when thyroid cancer cells progressed towards more malignant forms, they become more susceptible to HSV infection [19]. Another human herpesvirus that displays a marked tropism for thyroid follicular epithelial cells is Human Herpesvirus 6 A (HHV-6A) [20]. This virus is able to replicate in these cells and induce the expression of HLA class II antigens, facilitating NK-mediated killing. Moreover, a more efficient NK-mediated killing of HHV-6A-infected thyroid cells has been observed in HT patients compared to healthy donors, together with an increased T-cell response against HHV-6 U94 protein, suggesting a potential role for HHV-6A in triggering HT [20]. Regarding HHV-6A, another important topic that has been investigated for years is the relationship between viral infection and cancer. Despite the fact that the HHV-6A has been detected in some tumor types, which suggests a possible association of HHV-6A with cancer, its role in contributing to carcinogenesis remains an open question [21]. However, a more complete evaluation of this aspect should consider that HHV-6A could indirectly favor tumorigenesis through the cooperation with oncoviruses such as Epstein Barr virus (EBV) [22] or by promoting a long-lasting inflammation that is strictly linked to immune dysfunction and cancer [21]. Another possibility is that HHV-6A could trigger transformation by acting with a hit and run mechanism, as previously reported for other herpesviruses such as EBV [23], an effect that may lead to underestimation of cancers possibly associated with HHV-6A infection.

In the present study, we asked whether HHV-6A could infect the papillary thyroid carcinoma cells, BCPAP, and if viral infection could have onco-modulating effects in these cells, i.e., if it could lead to the acquirement of more malign properties. We focused on miRNAs expression and the release of cytokines involved in both inflammation and carcinogenesis, such as IL1 beta, VEGF, and IL-6. The latter is particularly important as, besides causing inflammation, it has been reported to sustain the growth of thyroid tumor cells. Genomic instability that leads to DNA mutation, the activation of oncogenes such as c-Myc, and the induction of features related to epithelial mesenchymal transition (EMT) are also evaluated in this study by comparing HHV-6A-infected BCPAP with uninfected control cells, as these effects could promote the progression of PTC towards more aggressive forms of thyroid tumors.

## 2. Materials and Methods

### 2.1. Cell Cultures, Infection, and Treatments

The papillary thyroid carcinoma cell line, BCPAP, was purchased from ECACC (Salisbury, UK) and maintained in culture in RPMI 1640 medium (Thermo Fisher Scientific, Waltham, MA, USA) supplemented with 10% fetal bovine serum (FCS) (Sigma-Aldrich, St. Louis, MO, USA), 10 IU/mL penicillin (Corning, Corning, NY, USA) and 10 μg/mL streptomycin (Corning) at 37 °C, 5% CO_2_ in a humidified tissue culture incubator [24].

HHV-6A was propagated in HSB-2 cells. The virus stock was obtained from a 7-day supernatant of infected cells, when more than 80% of the cells showed a cytopathic effect. Cell-free culture fluid was harvested, filtered through a 0.45-mm-pore-size filter, and pelleted by centrifugation at 25,000× *g* for 90 min at 4 °C. BCPAP cells were plated in 6-well plates at a density of 2 × 10^6^ cells each well and, the day after, were infected with an appropriate dilution of the HHV-6A viral stock (containing about 7 × 10^7^ DNA viral copies) in 0.5 mL. After 1 h at 37 °C, 1.5 mL of complete medium was added, and BCPAP cultures were maintained for 60 h. Mock control (CT) BCPAP cells were treated with 0.22 mm pore size filtered and heat inactivated virus stock.

### 2.2. Quantitative Real Time-Polymerase Chain Reaction (qRT-PCR)

To evaluate infection with HHV-6A, after 60 h, BCPAP cells were first washed with phosphate-buffered saline (PBS; Corning) then treated with 0.25% trypsin-EDTA to detach cells and with 1 u/μL DNaseI (Norgen Biotek Corp., Thorold, ON, Canada) for an additional 30 min at 37 °C to remove the non-internalized virus and extracellular viral DNA, respectively. Subsequently, DNA extraction was carried out by the ELITe Galaxy system (ELITechGroup S.p.A., Turin, Italy.) according to the manufacturer’s instructions. Extracted samples were then analyzed for the presence of HHV-6 DNA by quantitative TaqMan RT-PCR using a commercially available kit that amplifies a sequence relative to ORF 13R region, U67 gene (ELITechGroup S.p.A., Milan, Italy) using ABI 7300 real-time PCR System (Applied Biosystem, Waltham, MA, USA).

### 2.3. Cell Viability Assay

A Trypan blue dye exclusion assay was used to determine the number of viable cells. Cell suspension of HHV-6A- or mock-infected BCPAP was mixed with trypan blue at 1:1 ratio (Sigma-Aldrich, Burlington, MA, USA; cat. n. T8154), and live cells were counted via light microscopy using a Neubauer hemocytometer. Experiments were performed in triplicate and repeated at least three times.

### 2.4. FACS Analysis

BCPAP cells infected or mock-infected were washed with PBS 1X and incubated for 30 min at 4 °C with appropriate isotype control antibodies (Milteny Biotec, Bologna, Italy, 130-092-213) or mouse monoclonal antibody against gp110 (kindly provided by the HHV-6 Foundation). Cells were washed twice and then analyzed via FACSCalibur, using CELLQuest software (BD Biosciences, San Jose, CA, USA). Cells were gated according to their FSC and SSC properties. At least 10^4^ events were acquired for each sample.

### 2.5. RNA Extraction and RT-qPCR

HHV-6A or mock-infected BCPAP RNA were obtained by an automated Maxwell RSC-Promega extractor, using the Maxwell RSC miRNA Tissue Kit miRNA (CAT # AS1460, Promega, Sydney, Australia).

Retro-transcription was performed using a Taq-Man MicroRNA Reverse Transcription Kit. TaqMan Individual microRNA assays (Cat. N.4427975, Thermo Fisher Scientific) were used to assess expression of has-miR-9 (assay ID: 000583), hsa-miR-221 (assay ID: 000524), hsa-miR-222 (assay ID: 002276), hsa-miR-146a-5p (assay ID: 000468), hsa-miR-155-5p (assay ID: 002623), and U6 snRNA (assay ID: 001973).

QPCR was performed using an Applied Biosystems Vii A 7 Real-Time PCR (Thermo Fisher Scientific). The relative expression levels of miRNAs were quantified using the 2^∆∆Cq^ method after normalization for U6 expression, used as housekeeping. All procedures were performed according to the manufacturer’s instructions.

### 2.6. Western Blot Analysis

After treatments, HHV6A or mock-infected cells were harvested, washed, and lysed in RIPA buffer with 150 mM NaCl, 1% NP-40 (Calbiochem), 50 mM Tris-HCl, pH 8, 0.5% deoxcycholic acid (Sigma Aldrich, Burlington, MA, USA), 0.1% SDS, protease, plus phosphatase inhibitors. Protein concentration was measured and 10 μg of proteins were subjected to electrophoresis by using 4–12% NuPage Bis-Tris gels (Sigma Aldrich). Proteins were then transferred to nitrocellulose membranes, as already described [25]. Finally, after blocking PBS 0.1% Tween 20 solution containing 3% of BSA, blots were incubated with specific primary antibodies for 1 h at room temperature or overnight at 4 °C. After three washings in PBS-0.1% Tween 20 (washing solution), membranes were incubated for 30 min with a secondary antibody conjugated to horseradish peroxidase. Finally, membranes were washed three times with washing solution and then developed using ECL Blotting Substrate (Advansta, San Francisco, CA, USA).

### 2.7. Antibodies

To evaluate protein expression, the following antibodies were used: mouse monoclonal anti-pH2AX (Ser 139) (1:100) (Santa Cruz Biotechnology Inc., Dallas, TX, USA, sc-517348), rabbit monoclonal anti-Vimentin (1:500) (Cell Signaling, Danvers, MA, USA, 5741), rabbit monoclonal anti-PTEN (1:500) (Cell Signaling, Danvers, MA, USA, 9559), mouse monoclonal anti-p53 (1:100) (clone DO-1, Santa Cruz Biotechnology Inc., Dallas, TX, USA, sc-126), rabbit polyclonal anti-cMyc (1:1000) (Proteintech, Rosemont, IL, USA, 10828-1-AP), and rabbit monoclonal anti-Bcl-xL (1:500) (Cell Signaling, Danvers, MA, USA, 2764). Mouse monoclonal anti-β-actin (1:10,000) (Sigma Aldrich, A2228) was used as loading control. Goat anti-mouse IgG-HRP (1:10,000) (Bethyl Laboratories, Montgomery, TX, USA, A90-116P) and goat anti-rabbit IgG-HRP (1:10,000) (Bethyl Laboratories, Montgomery, TX, USA, A120-101P) were used as secondary antibodies.

### 2.8. Chemiluminescent Immunometric Assay (Luminex Assay)

Supernatants from infected or mock-control-infected BCPAP cells were collected and vascular endothelial growth factor (VEGF), interleukin-1 beta (IL-1β), interleukin-6 (IL-6), and cathepsin S release were measured via a magnetic Luminex assay, using a human pre-mixed multi-analyte kit (R&D systems Bio-Techne, LXSAHM, Minneapolis, MN, USA) according to the manufacturer’s instructions.

### 2.9. Densitometric Analysis

Densitometric analysis via Western blots was performed using the Image J software (1.47 version, NIH, Bethesda, MD, USA), which was downloaded from the NIH website (http://imagej.nih.gov (accessed on 10 February 2022)).

### 2.10. Statistical Analysis

Results are represented as the mean ± standard deviation (S.D.) of at least three independent experiments, and statistical analyses were performed with the Graphpad Prism^®^ software 10.0.3 (Graphpad software Inc., La Jolla, CA, USA). A student’s *t* test or a nonparametric one-way ANOVA test were used to demonstrate statistical significance. Difference was considered statistically significant when the *p*-value was: * < 0.05; ** < 0.01; *** < 0.001, and **** < 0.0001 (not significative—ns).

## 3. Results

### 3.1. HHV-6 Infection of BCPAP Cells Does Not Induce Cytopathic Effects and Dysregulates Expression of Several miRNAs

**Figure 1:**
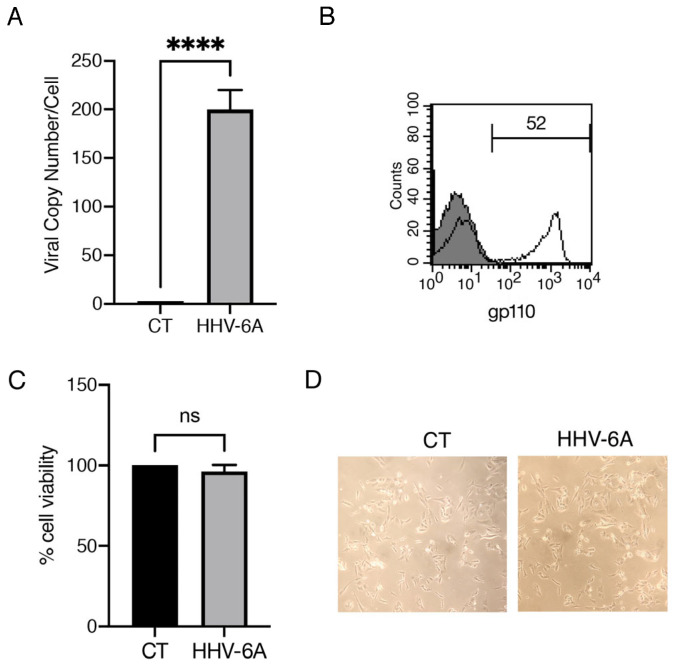
HHV-6A infects BCPAP without inducing a cytotoxic effect. (**A**) viral copy number determined by qRT-PCR in mock-control- (CT) and in HHV-6A-infected cells after 60 h of infection. (**B**) viral infection evaluated by FACS analysis analyzing the viral antigen gp100 expression following infection. (**C**) Cell viability evaluated by trypan blue exclusion assay. Histograms represent the percentage of viable cells ± SD. Data are the mean of three experiments. **** *p* < 0.0001. ns = not significant (**D**) Cellular morphology of mock- (CT) and HHV-6A-infected BCPAP cells (representative experiment, light microscopy ×40 magnification).

BCPAP cells were exposed to HHV-6A, originating from the supernatants of infected HSB2 (GS) cells, and the virus’s ability to infect BCPAP cells was evaluated via qRT-PCR. A high copy number was detected in infected cells, analysed 60 h post infection (Figure 1A). At this time the HHV-6A protein gp110 was also expressed by most of the infected cells, as evidenced by FACS analysis (Figure 1B). We then found that the virus did not affect cell survival compared to control cells (Figure 1C), and, accordingly, it did not cause a visible cytopathic effect in BCPAP-infected cells (Figure 1D).

**Figure 2:**
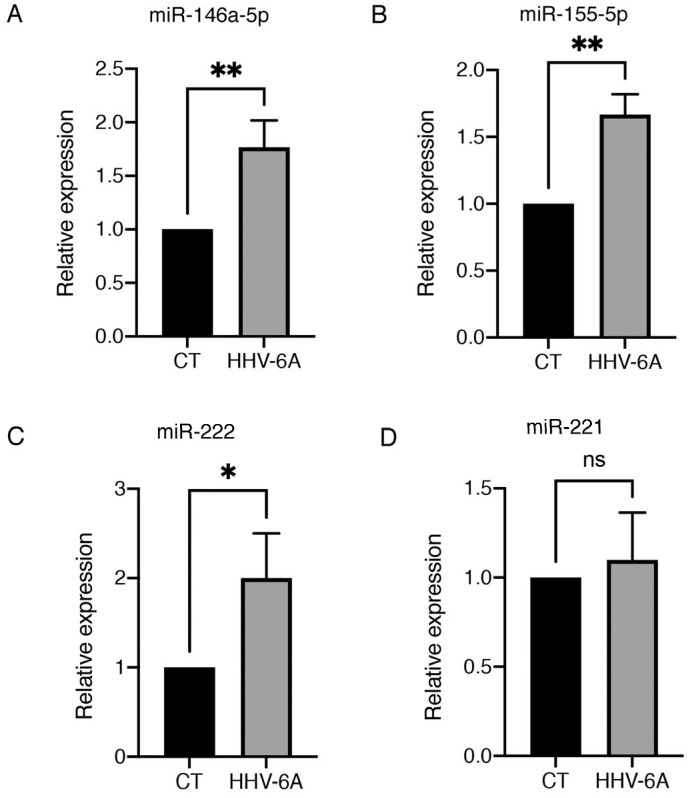
HHV-6A infection in BCPAP cells modulates the expression of miRNAs. Expression of (**A**) *miR-146a-5p,* (**B**) *miR-155-5p,* (**C**) *miR222*, and (**D**) *miR-221* in mock-control- (CT) or HHV-6A-infected BCPAP cells after 60 h from infection. Relative expression for each microRNA (miRNA) was normalized using *U6* small nuclear RNA (snRNA) housekeeping gene. Histograms represent the mean of three replicates ± S.D. *p* value: * *p* < 0.05; ** *p* < 0.01; ns, not significant.

As HHV-6A infection has been shown to alter miRNAs expression in thyrocytes, and based on the knowledge that the dysregulation of several miRNA is strongly involved in carcinogenesis, we evaluated if HHV-6A could alter the expression of miRNAs that could promote thyroid cancer progression in BCPAP cells. MiR-146a-5p, miR-155, miR-221, and miR-222 were evaluated via qRT-PCR in HHV-6A-infected and mock control cells. As shown in Figure 2, we found that the expression of 146a-5p, miR-155, miR-222 were upregulated after HHV-6A infection, which suggests that through this mechanism, the virus could favor the progression of PTC towards more aggressive forms of cancer.

### 3.2. HHV-6A Promotes the Release of Pro-Inflammatory Cytokines by Infected BCPAP Cells

**Figure 3:**
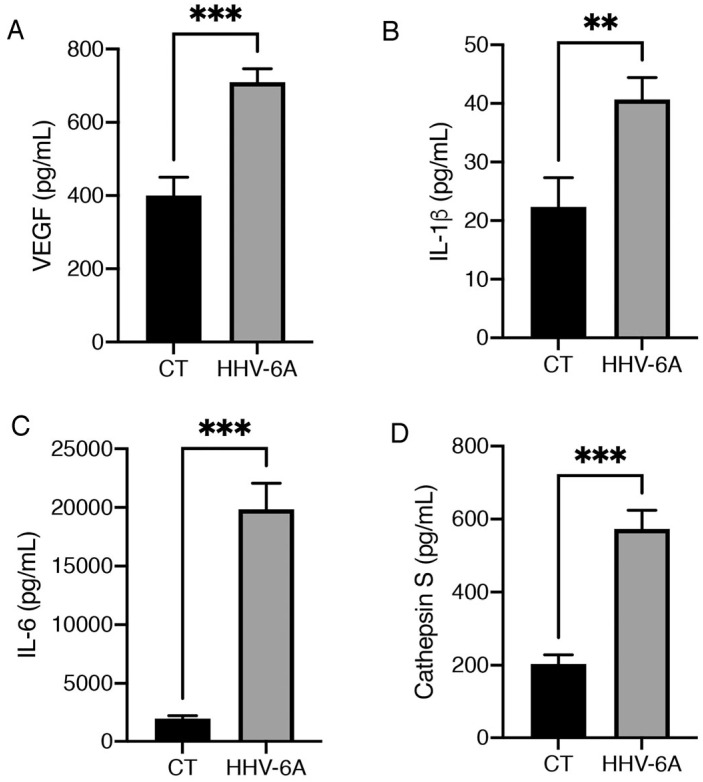
HHV-6A infection in BCPAP cells increases the release of pro-inflammatory cytokines. Detection of (**A**) VEGF, (**B**) IL-1β, (**C**) IL-6, and (**D**) Cathepsin S in supernatants from BCPAP cells infected with HHV-6A for 60 h and mock control BCPAP cells (CT), determined via multiplex ELISA. Histograms represent the mean ± SD of three independent experiments. *p* value: ** *p* < 0.01; *** *p* < 0.001.

The above-reported miRNAs can be activated via transcription factors that also promote pro-inflammatory cytokines release. The increase in pro-inflammatory cytokines could also contribute to papillary tumor progression [3], since inflammation is known to play a role in all steps of carcinogenesis, from cancer initiation to cancer progression [26]. Therefore, the production of cytokines involved in inflammation and carcinogenesis was evaluated in the supernatants of HHV-6A-infected BCPAP cells by using multiplex Elisa kits. The results shown in Figure 3A–C indicate that viral infection increased production of VEGF, which may exert various pro-tumorigenic effects such as invasiveness, vascularization and ability to metastasize [27] and IL-1 beta, that is also involved in tumor progression and immune escape [28]. Moreover, HHV-6A strongly induced the release of IL-6, cytokine reported to promote cancer progression [29], including that of thyroid tumors [30]. Finally, the release of cathepsin S, which is able to promote M2/TAM macrophage polarization [31], was found to be strongly increased by HHV-6A-infection (Figure 3D). Notably, the immune suppressive effect could also contribute to the high production of VEGF that HHV-6A infection also induces [32].

### 3.3. HHV-6A Increases Genomic Instability in BCPAP Cells and Up-Regulates PTEN/mutp53/c-Myc/Bcl-xL Axis and miR-9 to Promote EMT

**Figure 4:**
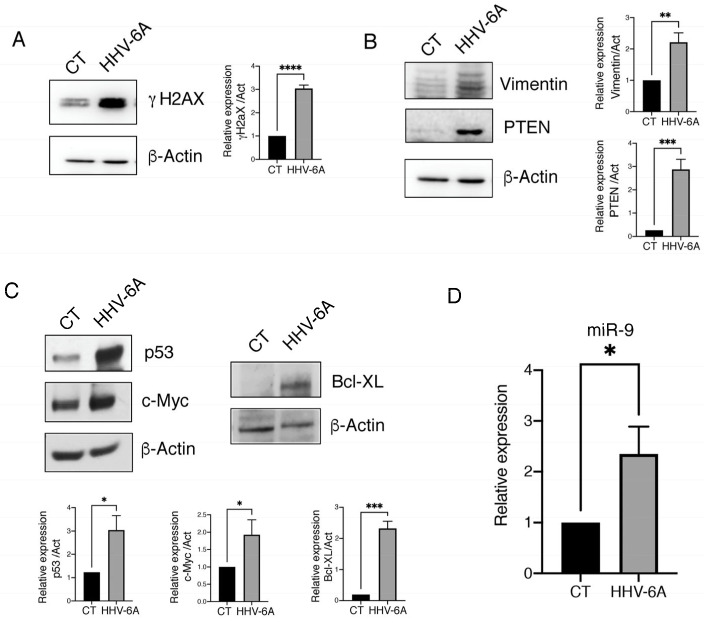
HHV-6A infection increases DNA damage and activates the PTEN/mutp53/c-Myc/Bcl-xL axis and miRNA9 to promote EMT. Expression of (**A**) γH2AX, (**B**) Vimentin and PTEN, (**C**) p53, c-Myc, and Bcl-xL via Western blot analysis in mock-control- (CT) or HHV-6A-infected BCPAP cells following 60 h of infection. β-Actin was used as loading control (representative experiment). Histograms represent densitometric analysis of the ratio between specific protein and β-Actin (mean of three different experiments). Data are represented as the mean ± S.D. *p* value: * < 0.05; ** < 0.01; *** < 0.001; **** < 0.0001. (**D**) *miR-9* expression evaluated by RT-qPCR in HHV-6A- and mock-infected (CT) BCPAP cells. The relative expression of *miR-9* was normalized using *U6* small nuclear RNA (snRNA) housekeeping gene. Histograms represent the mean of three replicates ± S.D. *p* value: * *p* < 0.05.

Genome instability, a key feature of malignant tumors involved in cancer progression, may be sustained by inflammation, ROS, and hypoxia [33]. Here, we found that HHV-6A infection increased DNA damage, as evaluated by the upregulation of the phosphorylated form of H2AX (γH2AX) in HHV-6A-infected BCPAP cells (Figure 4A), as its phosphorylation is mediated by the kinases such as ATM, ATR, and DNA-PK, which are able to sense DNA damage. We then investigated the possibility that HHV-6A could induce other changes related to tumor progression such as the acquirement of mesenchymal markers. This process, called EMT, could be promoted via the upregulation of the miR-221/222 cluster that is induced by HHV-6A. We found that the mesenchymal marker vimentin was up-regulated in BCPAP cells after HHV-6A infection (Figure 4B), and that, intriguingly, the expression of PTEN also increased (Figure 4B). Although PTEN may be mainly considered as an onco-suppressor gene in wtp53-carrying tumors, it has been reported to behave as an oncogene in mutp53-harboring cancer cells by increasing mutp53 expression and potentiating its oncogenic activity [34]. Here we found that concomitantly to PTEN, the expression of mutp53 increased in BCPAP cells, which are cells that are known to carry mutp53, and this effect was accompanied by the upregulation of c-Myc and BCL-xL (Figure 4C). These molecules are considered targets of mutp53 gain of function. Accordingly, a PTEN/mutant p53/c-Myc/Bcl-XL axis has been previously described in other mutp53-carrying cancer cell types such as glioblastoma cells [35].

Among other oncogenic properties, Myc may up-regulate several miRNAs, such as miR-9, which, in turn, can contribute to EMT [36]. As shown in Figure 4D, we found that miR-9 expression also increased in HHV-6A-infected BCPAP cells, another effect that may play a role in tumor progression.

## 4. Discussion

HHV-6A, a ubiquitous herpesvirus belonging to the β-subfamily, has been shown to be involved in the pathogenesis of Alzheimer’s disease [37] and to contribute to the onset of several autoimmune diseases [38], including Hashimoto thyroiditis [20,39]. Besides hematological and neuronal cells, the virus has indeed been reported to have a marked tropism for thyrocytes [40]. Regarding carcinogenesis, even if not directly, as demonstrated for the gamma herpes viruses EBV and KSHV [41,42,43], HHV-6A could indirectly contribute to this process, i.e., by creating an inflammatory/immune suppressive environment, due to the increased release of inflammatory/oncogenic cytokines and/or by activating miRNAs involved in cancer [21]. Indeed, immune dysfunction may prevent the control that immune system imposes on cancer onset, while inflammation can promote all steps of carcinogenesis, including progression towards more aggressive forms of cancer [44]. This may occur not only because pro-inflammatory and oncogenic pathways often overlap but also because inflammation induces tissue damage and repair attempts and is interconnected with the production of high levels of ROS. These molecules are able to promote genomic instability that may increase the probability that onco-suppressors, such as, for example, p53, or proteins involved in DNA damage repair pathways, such as BRCA1, undergo loss-of-function mutations [45]. In the present study, we found that HHV-6A was able to infect BCPAP, a cell line derived from well-differentiated papillary thyroid carcinoma (PTC), the most benign form of thyroid cancer, and induce several effects that could favor progression towards more aggressive forms of thyroid cancers such as FTC or ATC. The latter is the less differentiated form of thyroid cancer, the progression of which from PTC may be promoted by a permissive tumor microenvironment [3]. Interestingly, we found that HHV-6A altered the tumor microenvironment, dysregulating the production of several pro-inflammatory and pro-oncogenic cytokines by BCPAP cells; indeed, it increased the release of VEGF—that is, a pro-angiogenic, pro-tumorigenic, and immune-suppressive cytokine [27]—and IL-1 beta which, besides inflammation, may sustain carcinogenesis by contributing to angiogenesis, EMT, and shaping the tumor microenvironment into an immunosuppressive one [46]. Notably, HHV-6A infection very strongly stimulated the release of IL-6, which can be also considered a growth factor for thyroid cancer stem cells [46,47] and which has been shown to sustain thyroid cancer progression [30].

The expression of several miRNAs involved in oncogenesis was also up-regulated by HHV-6A infection in BCPAP cells. Some of these miRNAs, such as miR-9 and miR-221/222 cluster, have been reported to promote EMT [36], a feature that infected BCPAP cells acquire after infection, as suggested by the upregulation of the mesenchymal marker vimentin. HHV-6A infection also activated a PTEN/mutant p53/c-Myc/Bcl-XL axis in BCPAP, an axis previously described in other mutp53-carrying cancer cell types. The virus enhanced genome instability, which may be considered another important pro-oncogenic effect, correlated with inflammation and promoted by miRNAs, including miR-155-5p [48]. In conclusion, this study shows for the first time that PTC cells could be infected by the ubiquitous herpesvirus HHV-6A, already known to display a tropism for thyrocytes, and that infection can induce a variety of interconnected effects that are able to favor the progression of PTC towards more aggressive forms of thyroid cancers. Indeed, particularly important was the finding that HHV-6A was able to alter the tumor microenvironment, which is one of the key factors previously reported to promote thyroid cancer progression [3]. Therefore, targeting molecules such inflammatory cytokines, especially IL-6, whose production strongly increased following HHV6-A infection, may help to prevent inflammation-driven genome instability and possibly to counteract the up-regulation of PTEN and the interconnected activation of mutp53. Interestingly, mutp53 has been shown to promote the activation of STAT3 [49], and this pathway, which may, in turn, sustain mutp53 [50], is known to be strongly interconnected with IL-6 release [51]. Therefore, inhibition of IL-6 could be a promising strategy by which to reduce STAT3 phosphorylation in HHV-6A-infected BCPAP and may also help to interrupt the positive feedback loop that STAT3 establishes with mutp53—a criminal alliance that plays a pivotal role in carcinogenesis. Based on this and previous studies, it appears that HHV6-A may drive a multistep process of carcinogenesis in the thyroid, from autoimmune, Hashimoto-like thyroiditis to papillary cancer and to more undifferentiated and aggressive forms of cancer. Similar to other ubiquitous viruses that can be frequently detected in cancer biopsies but are not able to transform normal cells, HHV-6A could contribute to oncogenesis by playing an onco-modulatory effect. Such characteristics have been reported for other ubiquitous viruses, such as for HCMV, whose regulatory proteins can promote cancer cell proliferation, survival, invasiveness, and angiogenesis in tumor cells [52].

## Data Availability

The datasets generated and analyzed during the current study are available from the corresponding author upon reasonable request.
